# Organisational changes in service provision outside critical care impact on referral patterns

**DOI:** 10.1186/cc13198

**Published:** 2014-03-17

**Authors:** A Tridente, L Cecchini, S Lobaz, A Chick, S Keep, S Furmanova, D Bryden

**Affiliations:** 1 St Helens and Knowsley, Liverpool, UK; 2 SMH, Manchester, UK; 3 STH, Sheffield, UK; 4 UHW, Cardiff, UK

## Introduction

Demand for critical care (CC) resources is constantly increasing in the face of limited availability. Guidance for triage exists but may no longer reflect current practice [1,2]. We previously identified nonmedical and medical factors (comorbidities, physiological derangement and functional status) as predicting likelihood of admission of referred patients to CC [3-5]. Reduction in UK doctors' working hours and numbers has resulted in new ways of multidisciplinary teams working the hospital at night (H@N), which may have an impact on CC referral. We aimed to establish any effect ofhospital changes in service provision outside CC on the referral pattern and admissions.

## Methods

Data from prospectively enrolled urgent patient referrals were analysed comparing two periods: before (period 1: 2011/12) and after (period 2: 2013) the introduction of H@N. We collected data on acute physiological parameters, hospital length of stay, demographic and functional status, dependency and comorbidities. STATA was used for these preliminary analyses.

## Results

Comparing the two periods we found no significant differences in age, gender distribution, degree of acute physiological derangement, comorbidities, specialty of origin, time spent in hospital prior to referral and grade of referrer. By contrast, the proportion of out- of-hours referrals greatly increased (from 49.3% to 69.7%) along with the proportion of referrals deemed inappropriate for CC (from 37.3% to 54.8%); the proportion of patients accepted increased only marginally (from 46.7 to 50%) in the second period, compared with the first. Neither the number of beds available within the critical care department (*P *= 0.59) nor receiving the referral out of hours (*P *= 0.8) influenced the likelihood of admission. The factors predicting admission to CC (functional status, acute physiological derangement) did not differ significantly between the two periods examined. Housebound status was consistently found to be an independent predictor of admission refusal (OR 0.11, 95% CI 0.05 to 0.23, *P *< 0.001).

## Conclusion

Decision-making about admission to CC is based mainly on the assessment of patients' functional status. Organisational changes in the provision of healthcare services outside CC, such as H@N, have had a significant influence on CC workload and patterns of referrals.
